# Macrophage migration inhibitory factor regulates joint capsule fibrosis by promoting TGF-β1 production in fibroblasts

**DOI:** 10.7150/ijbs.57025

**Published:** 2021-04-29

**Authors:** Yuxin Zhang, Zhonglong Liu, Kexin Wang, Shenji Lu, Shuai Fan, Lili Xu, Bin Cai

**Affiliations:** 1 Department of Rehabilitation Medicine, Shanghai Ninth People's Hospital, Shanghai Jiao Tong University School of Medicine, Shanghai 200011, China.; 2 Shanghai Key Laboratory of Orthopedic Implants, Shanghai Ninth People's Hospital, Shanghai Jiao Tong University School of Medicine, Shanghai 200011, China.; 3 Department of Oral Maxillofacial & Head and Neck Oncology, Shanghai Ninth People's Hospital, Shanghai Jiao Tong University School of Medicine; College of Stomatology, Shanghai Jiao Tong University; National Center for Stomatology; National Clinical Research Center for Oral Diseases; Shanghai 200011, China.; 4 School of Kinesiology, Shanghai University of Sport, Shanghai 200438, China.

**Keywords:** macrophage migration inhibitory factor, inflammation, fibroblasts, transforming growth factor-β1, fibrosis

## Abstract

Joint capsule fibrosis caused by excessive inflammation results in post-traumatic joint contracture (PTJC). Transforming growth factor (TGF)-β1 plays a key role in PTJC by regulating fibroblast functions, however, cytokine-induced TGF-β1 expression in specific cell types remains poorly characterized. Macrophage migration inhibitory factor (MIF) is a proinflammatory cytokine involved in inflammation- and fibrosis-associated pathophysiology. In this study, we investigated whether MIF can facilitate TGF-β1 production from fibroblasts and regulate joint capsule fibrosis following PTJC. Our data demonstrated that MIF and TGF-β1 significantly increased in fibroblasts of injured rat posterior joint capsules. Treatment the lesion sites with MIF inhibitor 4-Iodo-6-phenylpyrimidine (4-IPP) reduced TGF-β1 production and relieved joint capsule inflammation and fibrosis. *In vitro*, MIF facilitated TGF-β1 expression in primary joint capsule fibroblasts by activating mitogen-activated protein kinase (MAPK) (P38, ERK) signaling through coupling with membrane surface receptor CD74, which in turn affected fibroblast functions and promoted MIF production. Our results reveal a novel function of trauma-induced MIF in the occurrence and development of joint capsule fibrosis. Further investigation of the underlying mechanism may provide potential therapeutic targets for PTJC.

## Introduction

Post-traumatic joint contracture (PTJC) is a major chronic musculoskeletal complication of joint injury and osteoarthritis 1-3, which seriously affects patients' quality of life and social productivity 2. Joint capsule fibrosis underlies the pathogenesis of PTJC, and is caused by inflammation, extracellular matrix (ECM) deposition, and collagen hyperplasia and arrangement disorder, together with other factors 3-5. As the main cell types in joint capsules, fibroblasts play a key role in ECM synthesis and remodeling, as well as inflammation and immune regulation 6. Under pathological conditions such as injury or inflammation, immune cells rapidly activate and secrete various cytokines, including transforming growth factor-β (TGF-β), interleukin (IL)-13, and platelet-derived growth factor (PDGF) 6-8. Secreted cytokines in turn promote fibroblast activation, proliferation, migration, differentiation into myofibroblasts expressing α-smooth muscle actin (α-SMA), and fibrotic factor production 5, 9. Modulation of these fibroblast events renders TGF-β1 a key regulator of joint capsule inflammation and fibrosis. TGF-β1 levels are chronically elevated in patients and animals with PTJC, and blocking TGF-β1 expression during joint trauma is an effective treatment strategy to reduce the severity of joint capsule fibrosis 10, 11. However, the mechanism underlying specific cytokine-induced TGF-β1 expression in distinct cell types during PTJC has not been fully elucidated.

Macrophage migration inhibitory factor (MIF) is a multifunctional proinflammatory cytokine ubiquitously distributed in various neurons, astrocytes, immune cells, endothelial cells, hepatocytes, and fibroblasts 12-14. In higher mammals, MIF regulates the pathophysiological processes of immunity, inflammation, wound healing, and cell proliferation and migration, and is therefore involved in numerous inflammation- and fibrosis-related diseases, including systemic infections, sepsis, osteoarthritis, rheumatoid arthritis, and atherosclerosis 15-17. Consequently, MIF has become a potential therapeutic target for these diseases. In chronic inflammatory diseases such as myocardial infarction, atrial fibrillation, and cystic fibrosis, MIF expression is increased, which promotes fibroblast proliferation and expression of fibrosis-related factors, especially TGF-β1 15, 18, 19. MIF exerts its physiological functions by interacting with membrane receptor CD74, which forms a receptor complex with chemokine receptors CXCR2, CXCR4, or CXCR7 to activate multiple intracellular signaling cascades 20. We previously found that the MIF/CD74 axis elicited the inflammatory response in astrocytes and joint capsule fibroblasts via activating mitogen-activated protein kinase (MAPK) signaling, thus regulating the inflammatory microenvironment of injured tissues 21, 22. We therefore hypothesized that TGF-β1-modulated pathological fibroblast functions may be associated with the activation of the MIF/CD74 axis, through which it regulates joint capsule fibrosis.

In the present study, we examined the relationship between the expression of MIF and TGF-β1 in injured posterior joint capsules following PTJC in rats. We further investigated the effect of MIF on TGF-β1 expression in joint capsule fibroblasts as well as the underlying molecular mechanism. Our results revealed that the MIF/CD74 axis affects pathological changes of joint capsule fibrosis by activating MAPK (P38, ERK) signaling to promote TGF-β1 expression in joint capsule fibroblasts during PTJC.

## Materials and Methods

### Animals

Male Sprague Dawley (SD) rats (6 weeks old, 180-220 g) were purchased from Shanghai SIPPR-Bk Lab Animal Co., Ltd. and maintained in specific pathogen-free laboratory animal facilities of the Ninth People's Hospital Affiliated to Shanghai Jiao Tong University School of Medicine. All rats were acclimated to the facility for seven days before *in vivo* experiments. All animal experiments complied with the National Institutes of Health Guide for the Care and Use of Laboratory Animals and were reviewed and approved by the Institution of Animal Care and Use Committee (IACUC) of the Ninth People's Hospital Affiliated to Shanghai Jiao Tong University School of Medicine. All animals were housed in individual cages under a 12-h light/dark cycle at room temperature (23 ± 1 °C) with free access to food and water.

### Establishment of rat PTJC model

The number of surgically treated animals was calculated as 6 per experimental group as shown in Figure S1. The PTJC model was established based on previous research 22, 23. Briefly, rats were anesthetized with an intraperitoneal injection of sodium pentobarbital (50 mg/kg), placed in supine position, and prepared for surgery under aseptic conditions. The fur was shaved from the surgical site of the right knee joint, and the skin was disinfected with chlorhexidine. A 15-mm midline skin incision was made, and a lateral parapatellar arthrotomy was performed. The patella was reflected medially and the femoral condyles were exposed. Two cortical windows 1.5-mm in diameter were made from the non-articulating cartilaginous regions of the medial and lateral femoral condyles using a 1.5-mm drill bit. The anterior/posterior cruciate ligament was sequentially incised and the knee joint hyperextended to -45° to disrupt the posterior capsule. The right knee joint was immobilized at approximately 135° of flexion with a 0.5-mm steel wire. The muscles and skin were sutured with silk threads after the patellofemoral joint reduction. For drug delivery, 10 μL of 100 mM 4-IPP (TOCRIS, Bristol, UK) (4-IPP group) or vehicle (Control group) was injected into the joint cavity. After the surgery, the rats received sodium salicylate (150 mg/kg) for pain control, and unrestricted daily activity in cages was permitted. Rats were euthanized at 0, 1, 3, 7, and 14 days, the internal fixation was removed, and the range of motion (ROM) of the knee joint extension was measured using a mechanical goniometer (arthrometer) after myotomies of the trans-articular muscles, as has been described previously 24. Briefly, the lateral femoral condyle acted as the center of rotation and was pinned, while the proximal femur and distal tibia were attached to the two arms of the arthrometer. During motion, the two femoral points were fixed while the distal tibia could accommodate for translation at the knee joint. The reproducibility to repeated measures was within ± 4°. Conditions of temperature (21 °C) and angular velocity (3°/sec) were standard. ROM measurements in flexion and extension were performed at the torques of 667 g/cm. All measurements were made within 15 min after euthanasia. Posterior joint capsules were collected after ROM measurement for subsequent pathological assays.

### Primary joint capsule fibroblasts culture

Primary joint capsule fibroblasts were isolated and cultured by tissue culture method according to previous study 25. Briefly, ten SD rats (male, 4 weeks old) were sacrificed by an anesthesia overdose and disinfected in 75% alcohol for 10 min. The posterior capsule tissues of rat knees were obtained and washed with minimum essential medium (MEM; Invitrogen, Carlsbad, CA, USA). Then, the joint capsule tissues were cut into small pieces using ophthalmic scissors and were placed in tissue culture plates containing Dulbecco's minimum essential medium (DMEM; HyClone, Logan, USA) supplemented with 10% fetal bovine serum (Gibco, Grand Island, NY, USA), 100 U/mL penicillin (Gibco), and 100 μg/mL streptomycin (Gibco) in a 5% CO_2_ incubator at 37 °C to obtain joint capsule fibroblasts. After 3-5 days, fibroblasts started to migrate from the sub-sections. The tissues were removed when the culture reached approximately 90% confluence. The medium was changed every two days. Primary joint capsule fibroblasts were cultured to passages 2 to 4 and identified by cells exhibiting a characteristic morphology and positive staining for the fibroblast marker vimentin (Abcam, Cambridge, UK, 1:1000) before used in subsequent experiments.

### Small interfering RNA (siRNA) transfection *in vitro*

The rat primary joint capsule fibroblasts were prepared as above-mentioned. When the cell density reached 30%-50%, cells were transfected with siRNA targeting CD74 (RiboBio, Guangzhou, China) in the presence of Lipofectamine 2000 (Invitrogen) according to manufacturer's instructions 21. Briefly, 250 μL Opti-MEM was used to dilute 5 μL Lipofectamine 2000 transfection reagent and 5 μL 20 μM CD74 siRNA, respectively. The diluted transfection reagent and siRNA were mixed and placed at room temperature for 15 minutes. The complex was then added to a 6-well plate containing 1.5 mL DMEM medium and gently mixed. The cell culture plate was placed in 5% CO_2_ incubator at 37 °C for 4-6 hours, and then replaced with the complete medium without antibiotics for another 48 hours for subsequent experiments. A siRNA with the same nucleotide composition but lacking sequence homology to the CD74 was also designed as negative control.

### Western blot

Tissue samples of the posterior joint capsule collected at 0, 1, 3, 7, and 14 days following injury or primary joint capsule fibroblasts treated with various drugs (MIF, TGF-β1, siRNA or inhibitors) were lysed in RIPA buffer (Beyotime, Shanghai, China) supplemented with 1 mM PMSF (Beyotime). Protein concentration in the supernatant was quantified using the Protein Assay Kit II (Bio-Rad, Inc., California, USA) to ensure equal loading. Western blots were performed as described previously 21. The relative intensities of protein bands were normalized to those of β-actin. The following antibodies were used: β-actin (ProteinTech, Wuhan, China, 1:5000), MIF (Abcam, 1:1000), TGF-β1 (Abcam, 1:1000), α-smooth muscle actin (α-SMA, Abcam, 1:1000), Collagen I (Abcam, 1:1000), CD74 (Santa Cruz, CA, 1:100), p-ERK/ERK (Cell Signaling Technology, Danvers, MA, 1:1000), p-P38/P38 (Cell Signaling Technology, 1:1000), p-JNK/JNK (Cell Signaling Technology, 1:1000). Secondary antibodies included Goat anti-Rabbit IgG (H+L) (DyLight 800 4X PEG) (Invitrogen, 1:20000) and Goat anti-Mouse IgG (H+L) (DyLight 680) (Invitrogen, 1:20000). The fluorescent signals were determined with an Odyssey imaging system (Li-Cor, Lincoln, NE, USA).

### Co-immunoprecipitation (Co-IP)

Cell lysates were harvested and centrifuged at 14,000 rpm for 20 min to remove the debris after primary joint capsule fibroblast treatment with 2 μg/mL MIF (ProSpec, USA) for 24 h. Protein concentration in the supernatant was quantified using the Protein Assay Kit II (Bio-Rad). For immunoprecipitation analysis, total cell lysates (500 μg) were precleared with protein A plus G-Sepharose (Beyotime) before incubation with specific antibodies at 4 °C, followed by addition of protein A plus G-Sepharose. After several washes, samples were boiled and analyzed by immunoblotting using anti-MIF or anti-CD74 antibody.

### Quantitative RT-PCR

RNA from different groups of primary joint capsule fibroblasts after MIF, TGF-β1, siRNA or inhibitors treatment were extracted using TRIzol (Invitrogen) according to the manufacturer's instructions. Next, 1 μg of total RNA per sample was used to synthesize cDNA using the Omniscript reverse transcription kit (QIAGEN, Dusseldorf, Germany), and qPCR was carried out using SYBR® Premix Ex Taq™ (Takara Bio, Dalian, China) on a real-time PCR system (Applied Biosystems). We normalized gene expression to *Gapdh* levels and calculated the relative levels using 2^-ΔΔCt^. All reactions were performed in triplicates. The primers were designed and synthesized by Sangon Biotech (Shanghai, China) as follows: *Gapdh*: forward primer 5ʹ-ACA GCA ACA GGG TGG TGG AC-3ʹ, reverse primer 5ʹ-TTT GAG GGT GCA GCG AAC TT-3ʹ; *Mif*: forward primer 5ʹ-CTT GGG TCA CAC CGC ACT TA-3ʹ, reverse primer 5ʹ-TCG CTC GTG CCA CTA AAA GT-3ʹ; *Cd74*: forward primer 5ʹ-CAT CGG GCT CAC AGG TTT GG-3ʹ, reverse primer 5ʹ-CTG GTG GCT CTG CTC TTG GC-3ʹ; *Tgf-β1*: forward primer 5ʹ-AGC AAC AAT TCC TGG CGT TAC-3ʹ, reverse primer 5ʹ-TGT ATT CCG TCT CCT TGG TTC A-3ʹ.

### Immunofluorescence

Following treatment with 2 μg/mL MIF in the presence or absence of 50 μM 4-IPP for 24 h, primary joint capsule fibroblasts were fixed in 4% paraformaldehyde with 0.1% Triton X-100 (Sigma, St Louis, MO, USA) and subsequently incubated with 4% goat serum to block nonspecific binding. Then, cells were incubated with anti-TGF-β1 (Servicebio, Wuhan, China, 1:100), anti-MIF (Abcam, 1:100), or anti-CD74 (Santa Cruz, 1:50) primary antibodies overnight. Fluorescent secondary antibodies of FITC-labeled goat anti-rabbit IgG (Sigma, 1:400) and Cy3-labeled goat anti-rabbit IgG (Sigma, 1:400) were used to visualize the corresponding subsets. Cells were then stained with 4,6-diamidino-2-phenylindole (DAPI; Sigma, 1:4000) and phalloidin (Abcam, 1:1000), followed by observation under a confocal fluorescence microscope (Leica, Germany) and semi-quantitatively analyzed with Image-J software (National Institutes of Health, USA). The fluorescence was scored and quantified with blinding.

### Histological observation

Knee joint tissue samples were collected from experimental models at different time points, fixed in 4% paraformaldehyde, decalcified in 10% EDTA, and then embedded in paraffin. The knee joint specimens were sagittally sectioned (5 μm) using a microtome, mounted on microscope slides, and processed with hematoxylin-eosin (HE) and Masson trichrome staining. Histopathological evaluation of knee posterior joint capsule for inflammatory cell infiltration and tissue fibrosis was performed in a blind fashion using semi-quantitative score: sublining layer lymphoid cell infiltration score (0-none to diffuse infiltration; 1-lymphoid cell aggregate; 2-lymphoid follicles; 3-lymphoid follicles with germinal center formation) was used for HE 26 and area of the blue-stained collagen fibers in Masson trichrome staining was analyzed with Image-J software. For immunohistochemical staining, the tissue sections were co-incubated with antibodies against MIF (Abcam, 1:100), TGF-β1 (Servicebio, 1:100), vimentin (Abcam, 1:1000), and CD74 (Santa Cruz, 1:50). The sections were further incubated with FITC-labeled goat anti-mouse IgG (Gibco, 1:400), Cy3-labeled goat anti-rabbit IgG (Sigma, 1:400), and DAPI (Sigma, 1:4000). Zeiss Axio Imager light microscope (Zeiss, Germany) was used for observing and imaging the samples. All images were taken from similar areas of the posterior joint capsule and semi-quantitatively analyzed with Image-J software. Three sequential specimens per rat in each group were measured. The tissue sections were scored and quantified with blinding.

### Cell viability assay

Primary joint capsule fibroblasts were seeded at a density of 2 × 10^4^ cells/well. Cells were treated with different concentrations of MIF (0-2.5 μg/mL) or TGF-β1 (ImmunoClone, Houston, USA, 0-100 ng/mL) for 24 h. Then, 10 μL of Cell Counting Kit-8 (CCK-8; Dojindo, Kumamoto, Japan) was added to the cells and incubated for an additional 2 h at 37 °C. A spectrophotometer was used to quantitatively measure cell viability by detecting absorbance of each well at 450 nm. The absorbance measurement was conducted with blinding. Assays were performed three times using triplicate wells.

### Sequencing of mRNA and bioinformatics analysis

Total RNA of primary joint capsule fibroblasts following treatment with 2 μg/mL MIF for 24 h or 48 h was extracted using the mirVana miRNA Isolation Kit (Ambion, Texas, USA) and then selected by RNA Purification Beads (Illumina, California, USA) for library construction and RNA-seq analysis. The library was constructed using the Illumina TruSeq RNA Sample Prep Kit v2 and sequenced by the Illumina HiSeq-2000 for 50 cycles. High-quality reads that passed the Illumina quality filters were used for sequence analysis. Sequencing outcomes were normalized with Reads Per Kilobase per Million mapped reads (RPKM). Differentially expressed genes (DEGs) were designated according to criteria of fold change > 2 and false discovery rate < 0.05 compared to the control. Gene functions were annotated by Blastx against the NCBI database or the AGRIS database with an E-value threshold of 10^-5^. Gene ontology (GO) classification was obtained by WEGO via GO id annotated using the Perl and R programs. The Kyoto Encyclopedia of Genes and Genomes (KEGG) pathways were assigned to the sequences using the KEGG Automatic Annotation Server (KAAS) online.

### Statistical analysis

All results were expressed as the mean ± standard deviation after analysis by the SPSS 22.0 statistical software (SPSS Inc., Chicago, IL, USA). Parametric data were analyzed by Student's t-test or one-way analysis of variance (ANOVA) followed by post-hoc Tukey's test to compare two groups. A level of *P* < 0.05 was considered statistically significant.

## Results

### Injury-induced MIF promotes TGF-β1 expression in fibroblasts of the posterior joint capsule following PTJC

First, we established the rat knee joint PTJC model (Figure S2A) and measured the knee passive extension ROM (Control group). The normal knee extension ROM was 159.2 ± 3.4°, but was reduced to 157.1 ± 1.2°, 151.8 ± 1.7°, 130.3 ± 2.9°, and 96.8 ± 2.0° at 1, 3, 7, and 14 days post-induction of PTJC, respectively (Figure S2B). Histological analyses of the posterior joint capsule revealed increased numbers and density of inflammatory cells (HE, *P* = 0.0002) (Figure S2C), along with collagen fiber hyperplasia (*P* = 0.0015) and disordered arrangement (Masson) (Figure S2D). These changes mirrored those found in humans and indicated that inflammation and fibrosis are closely related to PTJC.

**Figure 1:**
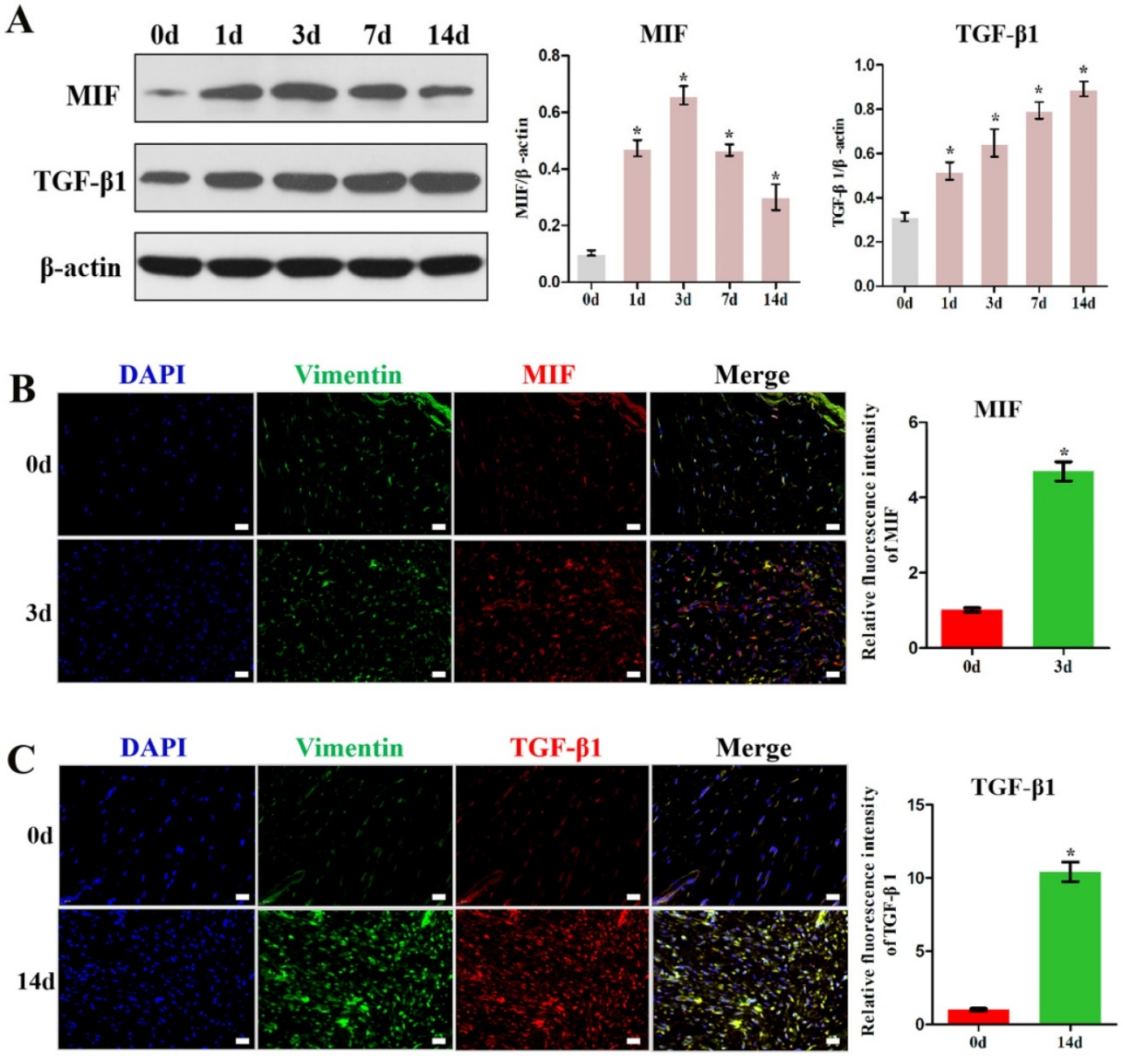
** MIF and TGF-β1 expression increased in the posterior joint capsule after PTJC. A,** Western blot analysis of MIF and TGF-β1 protein levels in posterior joint capsule following PTJC at 0, 1, 3, 7, and 14 days, respectively. Quantities were normalized to endogenous β-actin. **B,** Immunostaining showed colocalization of MIF (red) with fibroblasts in injured posterior joint capsule at 0 and 3 days after PTJC. Cell nucleus were stained with DAPI (blue). Vimentin was used as the marker of fibroblasts (green). **C,** Immunostaining showed colocalization of TGF-β1 (red) with fibroblasts in posterior joint capsule at 0 and 14 days after PTJC. Scale bars, 20 µm. All experiments were conducted independently at least three times. Error bars represent standard deviation. *P < 0.05 compared with 0 d group.

**Figure 2:**
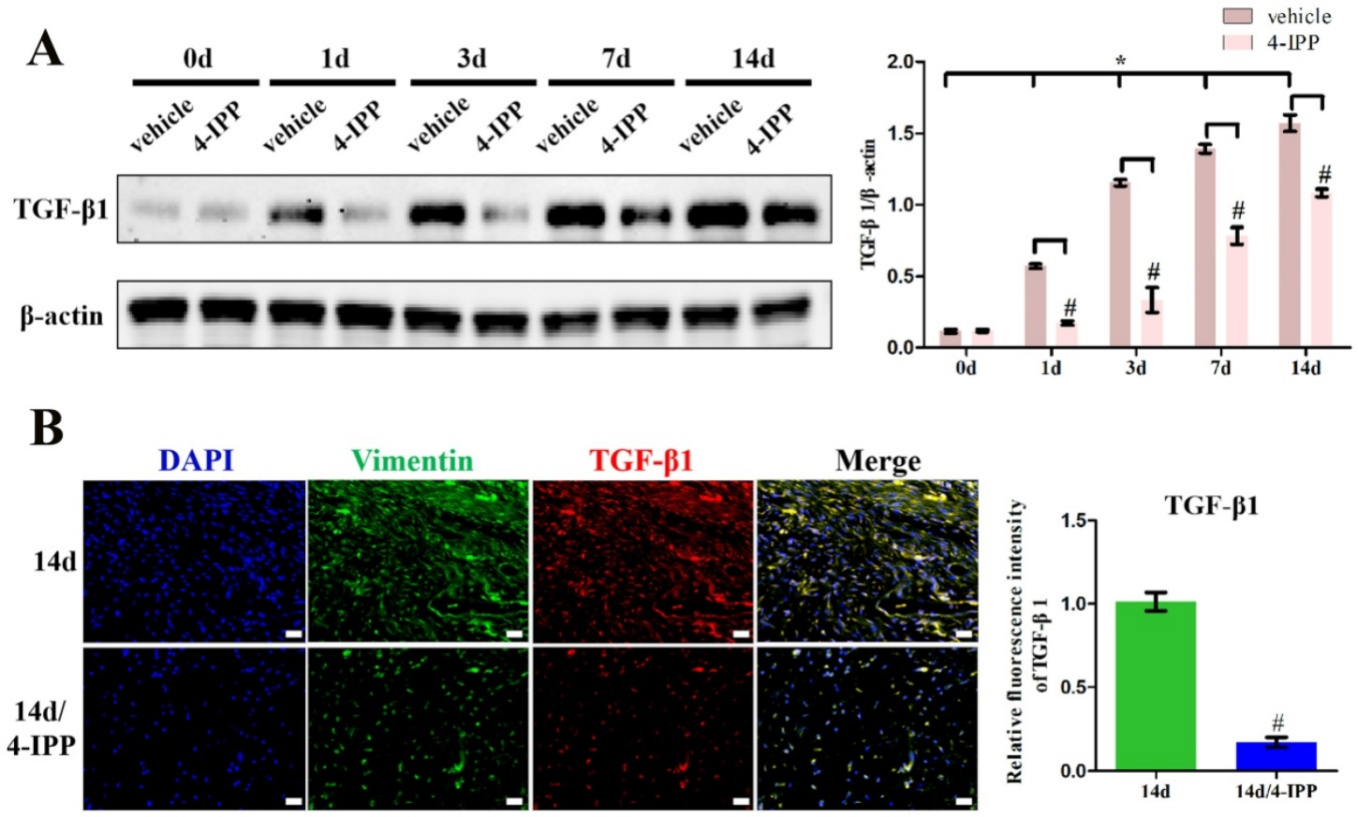
** Inhibition of MIF in the lesion area attenuated TGF-β1 expression. A,** Western blot analysis of TGF-β1 protein levels for the injured posterior joint capsule at different time points with or without injection of 10 µL 4-IPP (100 mM) at the lesion area by western blot. **B,** Immunostaining of TGF-β1 (red) in the injured posterior joint capsule at 14 days, with or without injection of 4-IPP, respectively. Scale bar, 20 µm. All experiments were conducted independently at least three times. Error bars represent standard deviation. *P < 0.05 compared with 0 d group. #*P* < 0.05 compared with vehicle group or 14 d group.

To examine whether MIF expression correlates with TGF-β1 production, we assayed the levels of these cytokines in the posterior joint capsule following PTJC at 0, 1, 3, 7, and 14 days (Control group) (Figure 1). Western blot analysis revealed that expression of MIF and TGF-β1 was induced in the posterior joint capsule following PTJC. MIF levels peaked on the third day (*P* < 0.0001) and TGF-β1 expression increased in a time-dependent manner (Figure 1A). To identify the specific cell types that respond to MIF, we performed immunostaining. MIF and TGF-β1 co-localized with vimentin-positive fibroblasts in the posterior joint capsule (Figure 1B-C). Consistent with the results of western blot, the fluorescence intensity of MIF and TGF-β1 was markedly enhanced after joint injury, indicating that the production of MIF and TGF-β1 was synchronous with the activation of fibroblasts. Injection of MIF inhibitor 4-IPP at the lesion sites significantly decreased the expression of MIF (*P* = 0.0002) (Figure S3A) and TGF-β1 in fibroblasts (Figure 2A-B), which attenuated joint capsule inflammatory cell infiltration (*P* = 0.0008) and collagen fiber hyperplasia (*P* = 0.0014) (Figure S3B-C). These data indicated that injury-induced MIF was able to facilitate TGF-β1 expression in fibroblasts of the posterior joint capsule following PTJC.

### MIF facilitates TGF-β1 expression in primary joint capsule fibroblasts *in vitro*

**Figure 3:**
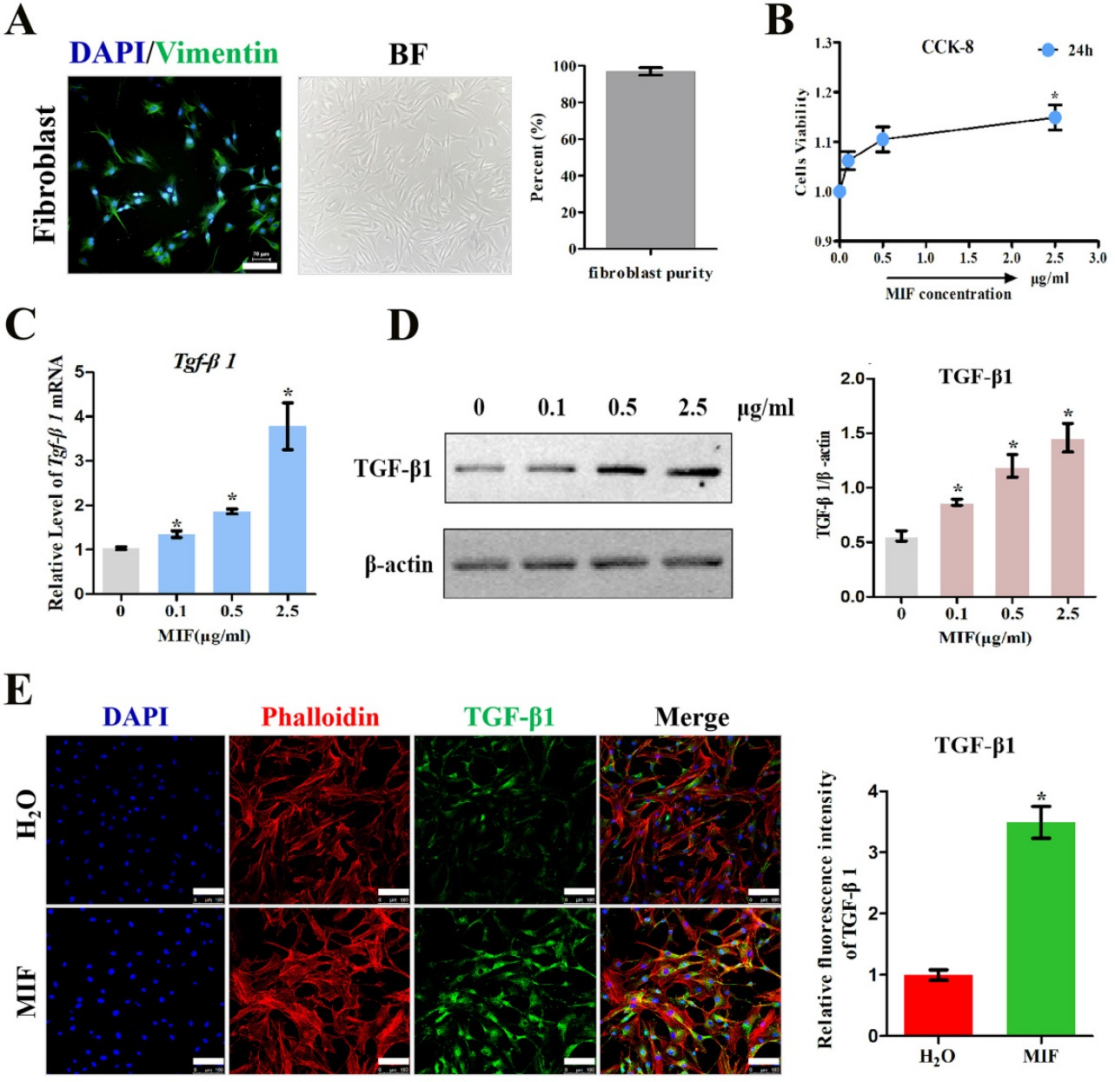
** Determination of TGF-β1 expression in response to MIF stimulation. A,** Isolated primary joint capsule fibroblasts stained with vimentin (green), DAPI (blue), and displayed in classical microscopy image (BF). **B,** CCK-8 assay was performed on joint capsule fibroblasts treated with varying MIF concentrations. **C-E,** Expression of TGF-β1 were assessed by qRT-PCR (**C**), western blot (**D**), and immunofluorescence (**E**, Phalloidin was used as cytoskeleton (red)) following joint capsule fibroblasts treatment with 0-2.5 µg/mL recombinant MIF for 24 h. Scale bar, 100 µm. All experiments were conducted independently at least three times. Error bars represent standard deviation. *P < 0.05 compared with 0 µg/mL group or H_2_O group.

**Figure 4:**
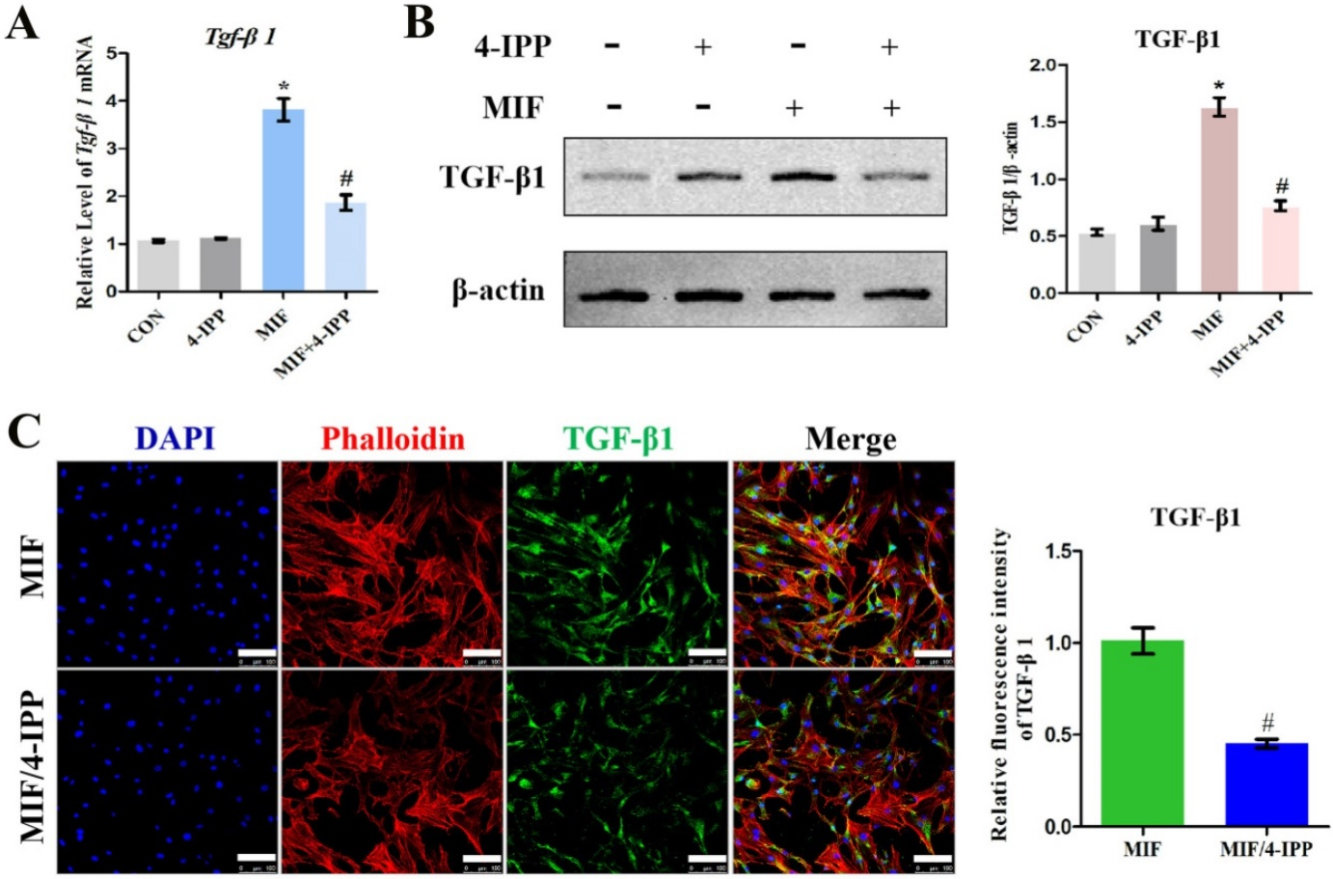
** Effects of MIF inhibitor 4-IPP on the expression of TGF-β1. A-C,** Expression of TGF-β1 in joint capsule fibroblasts in response to 2 µg/mL recombinant MIF combined with 50 µM 4-IPP treatment for 24 h was determined by qRT-PCR (**A**), western blot (**B**), and immunofluorescence (**C**). Scale bar, 100 µm. All experiments were conducted independently at least three times. Error bars represent standard deviation. *P < 0.05 compared with CON group. #*P* < 0.05 compared with MIF group.

To validate the role of MIF in regulating TGF-β1 expression in fibroblasts, primary joint capsule fibroblasts were cultured (Figure 3A) and treated with 0-2.5 μg/mL recombinant MIF for 24h. As shown by CCK-8, MIF slightly enhanced cell viability (*P* = 0.0092) (Figure 3B), indicating that this concentration range was applicable to subsequent experiments. We then detected the expression of TGF-β1 at both the transcriptional and translational levels. qRT-PCR and western blot demonstrated that TGF-β1 significantly increased in MIF-treated joint capsule fibroblasts in a dose-dependent manner (Figure 3C-D). In addition, the fluorescence intensity of TGF-β1 was markedly enhanced in joint capsule fibroblasts after 2 μg/mL MIF treatment (*P* = 0.0008) (Figure 3E). Addition of 50 μM 4-IPP to the cell culture efficiently blocked the effects of 2 μg/mL MIF on TGF-β1 expression (Figure 4). These results indicated that MIF could induce TGF-β1 expression in joint capsule fibroblasts.

### MIF regulates TGF-β1 expression in fibroblasts through membrane receptor CD74

**Figure 5:**
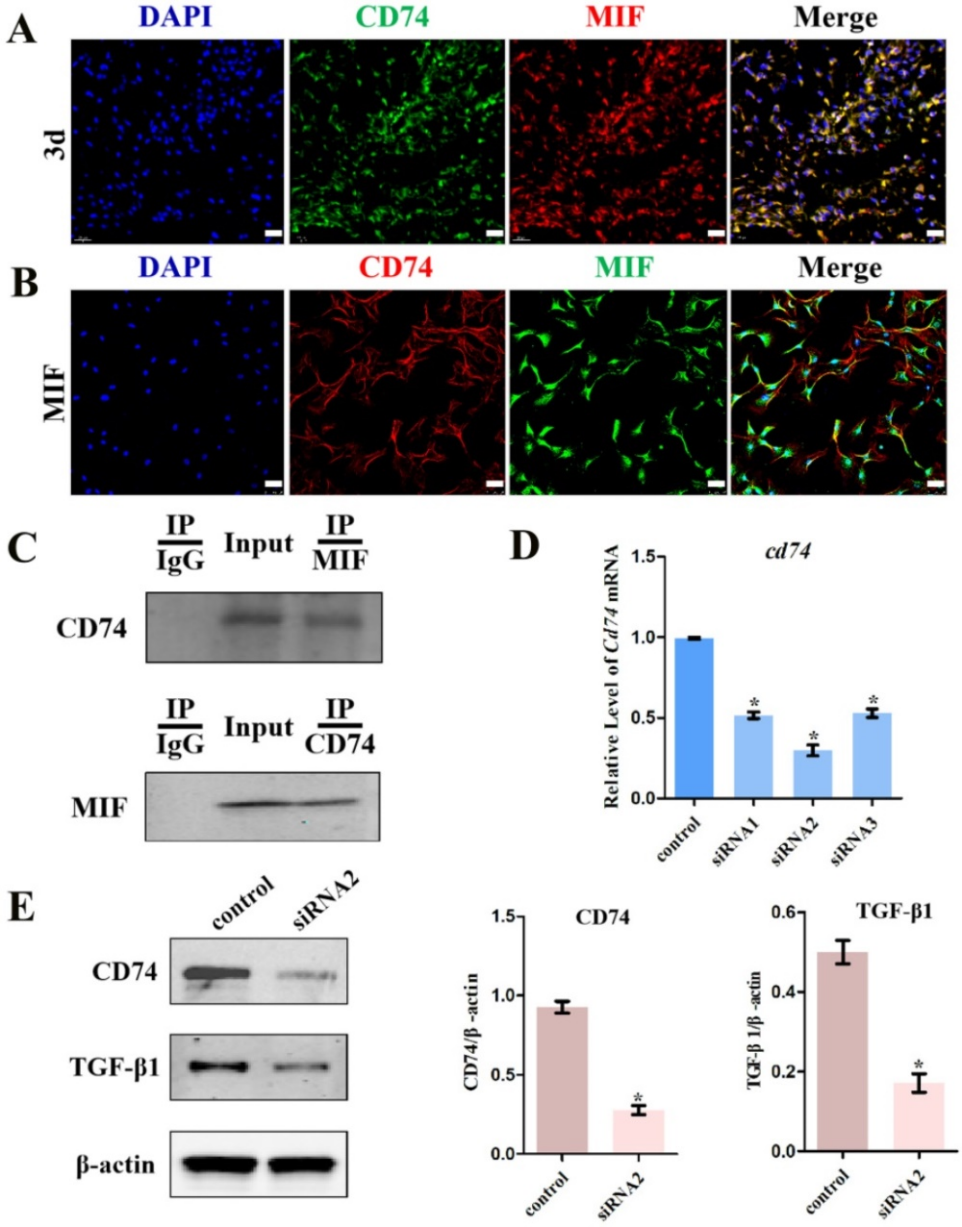
** Effects of CD74 on the MIF-induced TGF-β1 expression. A,** Colocalization of CD74 (green) and MIF (red) in the posterior joint capsule following PTJC at 3 days. Scale bar, 20 µm. **B,** Colocalization of CD74 (red) and MIF (green) in joint capsule fibroblasts after 2 µg/mL recombinant MIF treatment. Scale bar, 50 µm. **C,** Immunoprecipitation determined the interaction of MIF with CD74 receptor in joint capsule fibroblasts after 2 µg/mL MIF treatment. **D,** Knockdown efficiency of CD74 siRNA in joint capsule fibroblasts were tested by qRT-PCR, and siRNA2 was chosen for the subsequent experiments. **E,** Western blot analysis of CD74 and TGF-β1 expression following siRNA2 knockdown of CD74 receptor in joint capsule fibroblasts for 48 h, and then treated with 2 µg/mL MIF for 24 h. A siRNA (control) with the same nucleotide composition as siRNA2 but lacking sequence homology to the CD74 was also designed as negative control. All experiments were conducted independently at least three times. Error bars represent standard deviation. *P < 0.05 compared with control group.

Next, to clarify whether MIF interacts with CD74 in joint capsule fibroblasts and whether this interaction is associated to MIF-induced TGF-β1 production, we performed immunofluorescence and Co-IP using anti-MIF and anti-CD74 antibodies. Immunofluorescence demonstrated that the membrane receptor colocalized with MIF in the posterior joint capsule following PTJC (Figure 5A) and in joint capsule fibroblasts after MIF treatment (Figure 5B). As shown by Co-IP, CD74 was detected in the MIF-associated complexes after MIF treatment of joint capsule fibroblasts. Conversely, MIF was also co-precipitated in CD74-associated complexes (Figure 5C), indicating that MIF could bind to the CD74 receptor of joint capsule fibroblasts. Subsequently, we synthesized and evaluated three specific siRNA targeting CD74 to knockdown its endogenous expression 21, 22. SiRNA2, showing the highest interference efficiency, was selected for subsequent experiments (Figure 5D). Joint capsule fibroblasts were transfected with siRNA2 for 48 h, followed by treatment with 2 μg/mL MIF for 24 h. Western blot analysis of the cell lysate showed that CD74 silencing resulted in a significant decrease in TGF-β1 expression at the protein level (*P* = 0.0009) (Figure 5E). These findings suggested that MIF-induced TGF-β1 expression in joint capsule fibroblasts was dependent on the CD74 membrane receptor.

### MIF promotes TGF-β1 production through P38 and ERK signaling pathways

**Figure 6:**
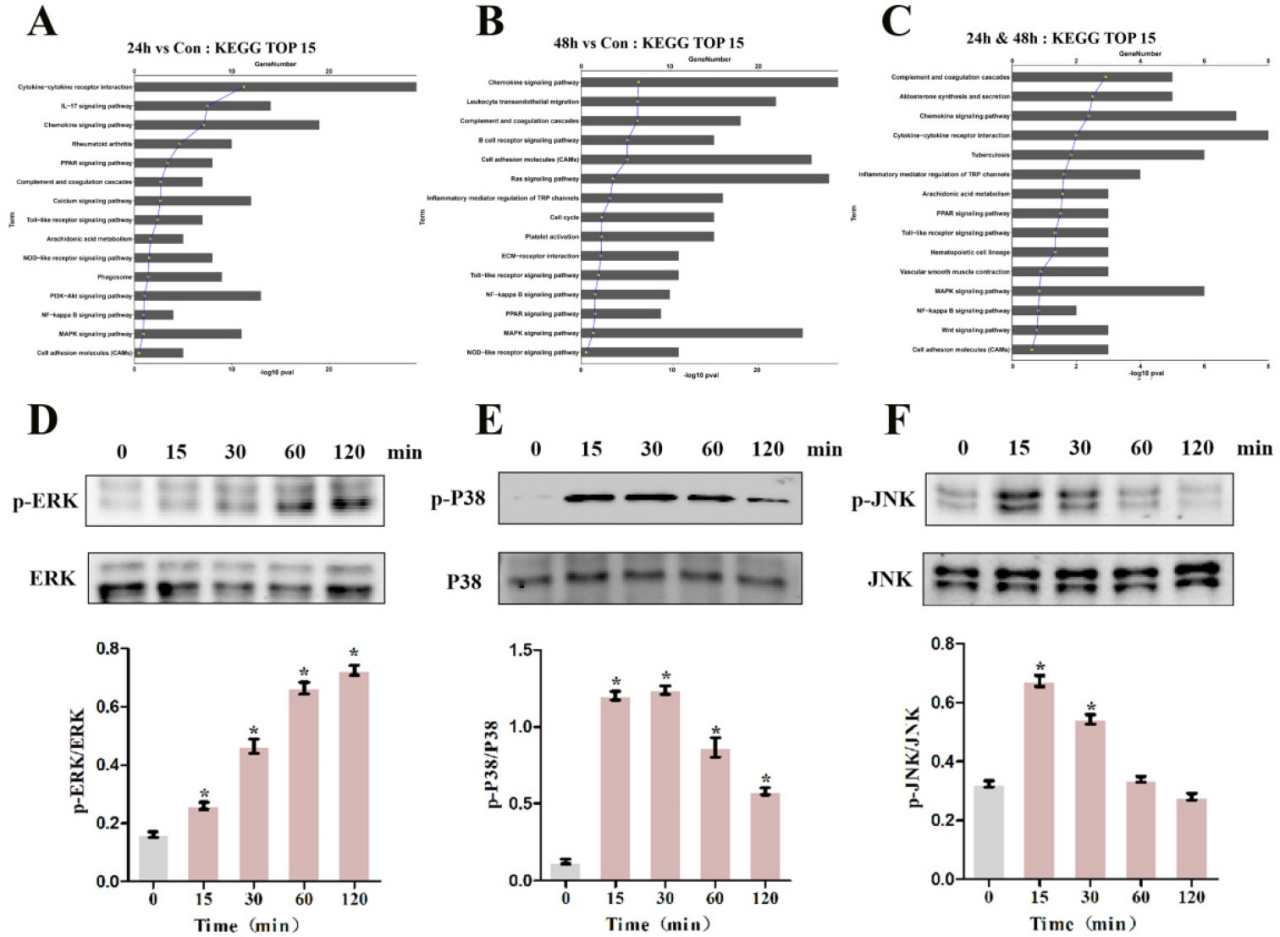
** Effects of MIF on the activation of intracellular signaling in joint capsule fibroblasts. A and B,** Top 15 most significantly enriched groups for the DEGs related to pathways following joint capsule fibroblast treatment with 2 µg/mL recombinant MIF for 24 h (**A**) or 48 h (**B**). **C,** Top 15 most significantly enriched groups for integrated DEGs (24 h & 48 h) related to pathways. **D-F,** Western blot analysis of MAPK signaling-related proteins p-ERK (**D**), p-P38 (**E**), and p-JNK (**F**) after joint capsule fibroblasts treated with 2 µg/mL recombinant MIF for 0, 15, 30, 60, and 120 min, respectively. All experiments were conducted independently at least three times. Error bars represent standard deviation. *P < 0.05 compared with 0 min group.

**Figure 7:**
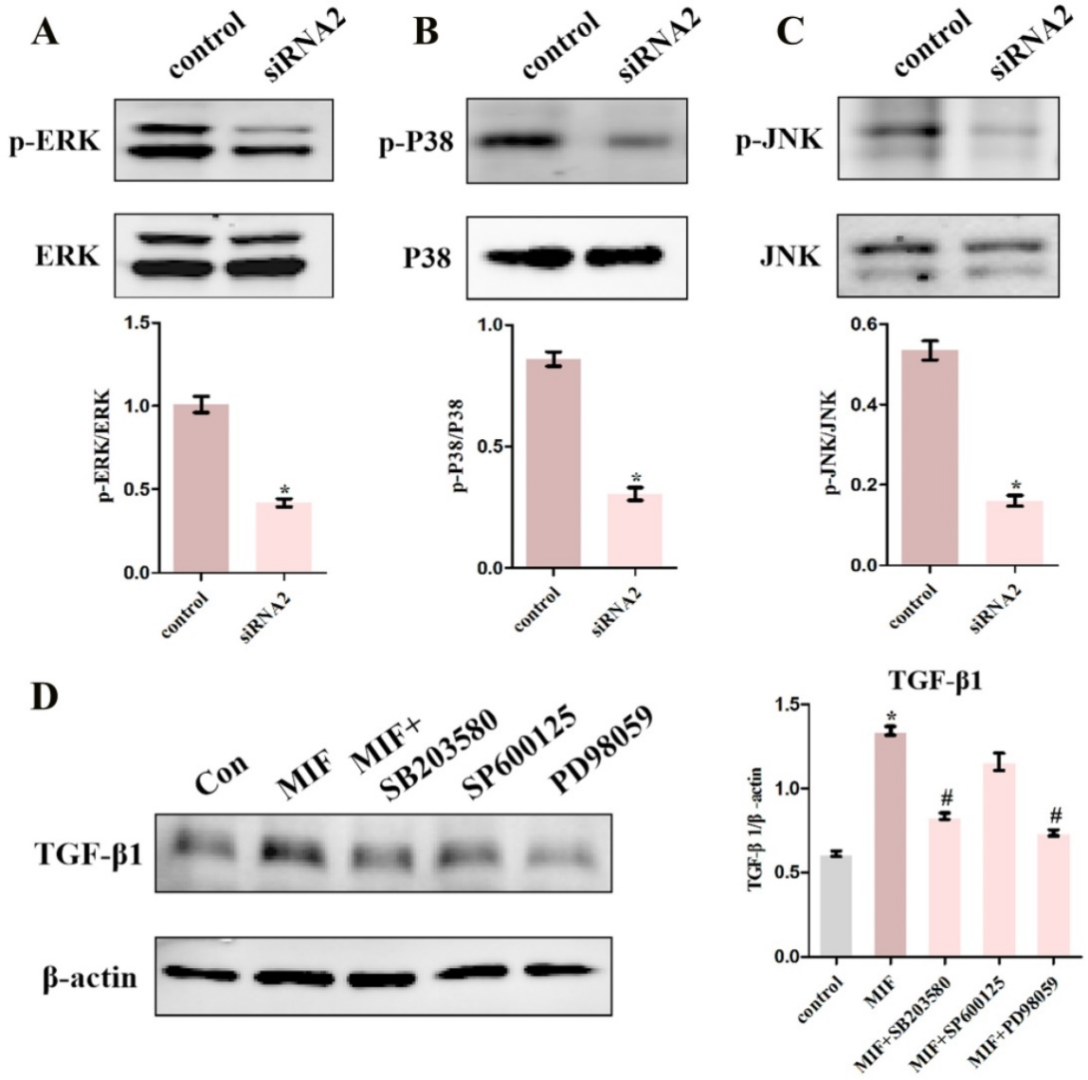
** Determination of intracellular signaling associated with regulation of TGF-β1 expression. A-C,** Western blot analysis of p-ERK (**A**), p-P38 (**B**), and p-JNK (**C**) following joint capsule fibroblasts treated with siRNA2 or control siRNA for 48 h and then with 2 µg/mL recombinant MIF for 30 min. **D,** Joint capsule fibroblasts were pretreated with 10 µM inhibitor of P38 (SB203580), JNK (SP600125), or ERK (PD98059) for 1 h before treatment with 2 µg/mL recombinant MIF for 24 h, western blot assayed TGF-β1 expression. All experiments were conducted independently at least three times. Error bars represent standard deviation. *P < 0.05 compared with control group. #*P* < 0.05 compared with MIF group.

To pinpoint the relevant pathways through which MIF regulates TGF-β1 production, we performed transcriptome sequencing analysis on primary joint capsule fibroblasts treated with 2 μg/mL recombinant MIF for 24 and 48 h. Then, KEGG function enrichment analysis was performed for all the DEGs at the two time points (Figure S4, S5). KEGG pathway analysis revealed that chemotactic signaling, inflammatory response, and related MAPK cascades were included in the top 15 significantly enriched functional pathways (Figure 6A-C). Consistent with transcriptome analysis, western blot showed that phosphorylation of MAPK signaling components (ERK, P38, and JNK) was rapidly induced 15 min after stimulation of joint capsule fibroblasts with 2 μg/mL recombinant MIF (Figure 6D-F). The MAPK pathway is a crucial intracellular signaling cascade that mediates MIF function 27, 28. To determine whether expression of TGF-β1 by MIF was associated with MAPK, we silenced the CD74 receptor in joint capsule fibroblasts and treated them with 2 μg/mL recombinant MIF for 30 min. Knockdown of CD74 led to a significant decrease in ERK, P38, and JNK phosphorylation levels (*P* = 0.0004; *P* = 0.0001; *P* = 0.0002) (Figure 7A-C). Subsequently, joint capsule fibroblasts were treated with inhibitors of P38 (SB203580, TOCRIS), JNK (SP600125, TOCRIS), and ERK (PD98059, TOCRIS) in the presence of 2 μg/mL recombinant MIF. Interestingly, TGF-β1 production was exclusively attenuated by treatment with ERK and P38 inhibitors (*P* = 0.0001;* P* <0.0001), rather than by the JNK inhibitor (*P* = 0.0792) (Figure 7D). These data indicated that MIF-induced TGF-β1 production in joint capsule fibroblasts was mainly regulated by ERK and P38 pathways.

### High TGF-β1 concentrations mediate and enhance the pro-fibrotic function of MIF

**Figure 8:**
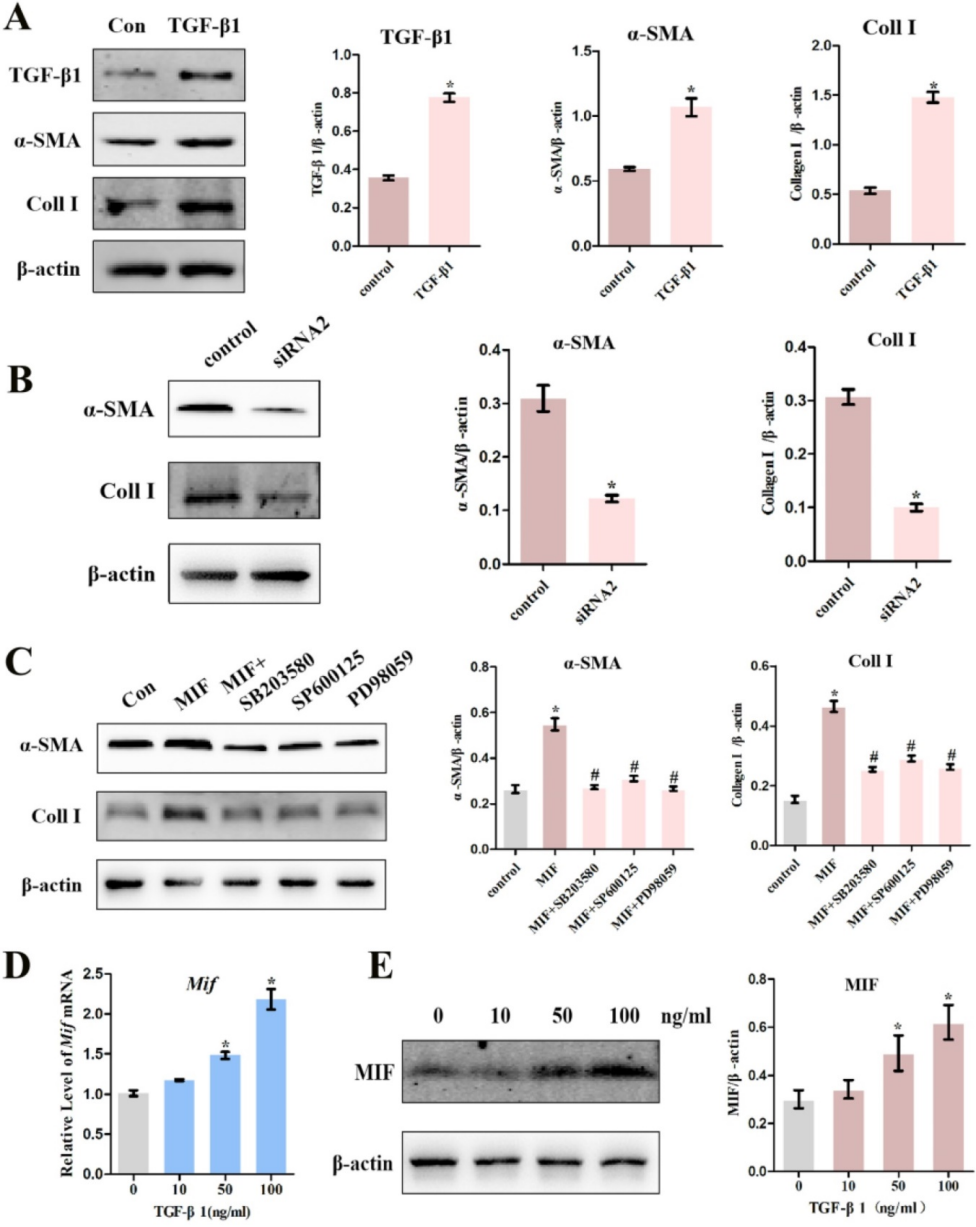
** Effects of TGF-β1 on the expression of pro-inflammatory and pro-fibrotic molecules. A,** Western blot analysis the expression of TGF-β1, α-SMA, and Collagen I in joint capsule fibroblasts following treatment with 10 ng/mL recombinant TGF-β1 for 24 h. **B and C,** Western blot analysis of α-SMA and Collagen I expression following siRNA2 knockdown of CD74 receptor for 48 h (**B**) or incubation with 10 µM inhibitor of P38 (SB203580), JNK (SP600125), or ERK (PD98059) for 1 h (**C**), and then treated joint capsule fibroblasts with 2 µg/mL recombinant MIF for 24 h. **D and E,** Expression of MIF were assessed by qRT-PCR (**D**) and western blot (**E**) following joint capsule fibroblasts treatment with 0-100 ng/mL recombinant TGF-β1 for 24 h. All experiments were conducted independently at least three times. Error bars represent standard deviation. *P < 0.05 compared with control group or 0 ng/mL group. #*P* < 0.05 compared with MIF group.

A large number of studies have shown that TGF-β1 can promote the differentiation of fibroblasts into myofibroblasts, excessive collagen production, and ECM protein accumulation 11. Consistent with this, we confirmed that 10 ng/mL TGF-β1 promotes the expression of myofibroblast marker α-SMA (*P* = 0.0026), collagen I (*P* = 0.0001), as well as its own expression (*P* = 0.0021) in activated joint capsule fibroblasts (Figure 8A). Moreover, we also found that MIF could directly induce α-SMA and Collagen I production in joint capsule fibroblasts and CD74 silencing (*P* = 0.0028; *P* = 0.0006) (Figure 8B) or MAPK pathway inhibition (Figure 8C) blocked this effect, which was synchronized with the changes in TGF-β1 in Figure 5E and Figure 7D. In a study, it was found that growth factors such as TGF-β can significantly upregulate the expression of MIF in mouse osteoblast cell lines 29. To explore whether TGF-β1 could also regulate MIF, we stimulated joint capsular fibroblasts with different concentrations of TGF-β1 for 24 h. As shown in Figure S6, TGF-β1 treatment did not affect cell viability. However, 50 and 100 ng/mL TGF-β1 significantly induced the expression of MIF in joint capsule fibroblasts (Figure 8D-E). These data indicated that when MIF-induced release of TGF-β1 accumulation to certain concentrations could lead to MIF production, thus forming a positive feedback loop promoting MIF-induced inflammation and fibrosis during PTJC.

## Discussion

PTJC is the main musculoskeletal disease caused by trauma or surgery and is characterized by irreversible loss of joint motion 1, 30. The development of PTJC is a complex pathological process accompanied by structural changes in the joints and surrounding tissues. Among these, posterior joint capsule fibrosis is the key anatomic factor underlying PTJC 3-5. Joint trauma triggers edema, bleeding, and inflammatory response, during which the resident and infiltrating immune and stromal cells are rapidly prompted to release various cytokines and chemokines 4, 6, 31. These factors converge into a complex network to jointly regulate the fibrosis process. Activated fibroblasts, as key cell mediators, advance this process through hyperproliferation, differentiation into myofibroblasts, regulation of ECM components, and inflammation 32. In addition, fibroblasts constitutively express a series of innate immune receptors and are therefore highly sensitive to infections or tissue damage 33-35. TGF-β1 is considered the most critical cytokine promoting fibrosis by affecting fibroblast functions 36-38. Despite the fact that TGF-β1 is upregulated in animal PTJC models and its inhibition can relieve joint capsule fibrosis 10, 11, 39, 40, the cellular source and the precise mechanisms orchestrating TGF-β1 production in fibrotic areas are not yet well understood.

Some studies reported that several different cell types such as platelets, macrophages, monocytes, neutrophils, T lymphocytes, and fibroblasts have been implicated in TGF-β1 production 3-7. In this study, we found that the expression of TGF-β1 was significantly increased in fibrotic joint capsule and co-localized with fibroblasts. Fibrosis develops as a complication of the inflammatory process. Acute inflammation is initiated by immune cells and persist only for a short time. Macrophages and other inflammatory cells decreased with the acute inflammation subsided 4-6, 41. Considering the fact that fibroblasts are the main cellular components of connective tissue, which participate in the regulation of acute and chronic inflammation 6 and the sustained increase of TGF-β1 is not affected by the regression of acute inflammation, we believe that joint capsule fibroblasts may be the main and sustained source of TGF-β1 during PTJC. Additionally, given that the TGF-β1 signaling contributes to exceptionally broad range of fundamental biological activities such as immunomodulation, fracture repair, and wound healing, upstream mediators of excessive TGF-β1 synthesis and signal transduction may be more promising targets for post-traumatic antifibrotic therapy.

Several cytokines have been reported to be responsible for inducing TGF-β1 expression in immune cells through multiple signaling pathways 6, 8, 31. MIF is constitutively expressed in various immune and non-immune cells of different histogenetic origins and can be induced by injury signals 13, 14. MIF shows a remarkable functional diversity, ranging from promoting pattern recognition receptor TLR-4 expression, enhancing inflammatory cell recruitment, and inhibiting p53-mediated cell apoptosis to eliminating the anti-inflammatory effects of glucocorticoids 12-14. Given such broad activities, it is not surprising that aberrant expression of MIF is implicated in several diseases. While previous studies on MIF-mediated chronic inflammation and fibrosis were mainly focused on the skin and different organs 15, 18, 19, its role in PTJC has not been previously reported. In the current study, we showed that MIF was induced in the posterior joint capsule after PTJC and that increased MIF activated joint capsule fibroblasts and promoted TGF-β1 expression, providing a novel mechanism for joint capsule fibrosis following PTJC.

MIF plays a pivotal role in regulating fibrosis of visceral organs such as the heart, liver, and lung by interacting with its high-affinity membrane receptor CD74 42-44. Similarly, in this study, we similarly found that the MIF/CD74 axis can activate joint capsule fibroblasts by promoting TGF-β1 expression, indicating the conserved regulatory mechanism of MIF in different cell types. Numerous signaling pathways are involved in tissue fibrosis, including MAPK, NF-κB, TGF-β1/SMAD, and Wnt/β-catenin signaling 45. To unveil the mechanism by which MIF induces TGF-β1 expression, we performed transcriptome analysis on MIF-treated joint capsule fibroblasts. KEGG pathway analysis revealed significant enrichment of the MAPK pathway, the main intracellular signal transduction pathway regulating cell proliferation, differentiation, survival, and apoptosis 46. The ERK signaling pathway is involved in urethral plate fibroblasts proliferation, apoptosis, and regulation of the cell cycle 47. Phosphorylation of P38 and JNK promotes proliferation of lung mesenchymal fibroblasts 48. We previously found that the MIF/CD74 axis regulates the inflammatory microenvironment of injured tissues via activating MAPK signaling (P38, JNK, ERK) in astrocytes 21. In the present study, we revealed a novel role of the MIF/CD74 axis in facilitating TGF-β1 production in joint capsule fibroblasts through activation of ERK and P38 signaling. Considering that MIF could activate joint capsule fibroblasts through all three pathways of MAPK, which suggested that MIF had a broader biological role in joint capsule fibrosis except for inducing TGF-β1 production.

TGF-β1 is an effective fibroblast activator and mitogen, as well as a key signal for fibroblast differentiation and collagen synthesis 49, 50. Consistent with these studies, we illustrate that treatment of joint capsule fibroblasts with TGF-β1 not only upregulated the levels of α-SMA and collagen I, but also the levels of TGF-β1 itself. This may be the reason why TGF-β1 expression increased in a time-dependent manner after PTJC, although MIF expression reached its peak on the third day. Considering that MIF/CD74 axis induced α-SMA and Collagen I production was synchronized with the changes in TGF-β1, TGF-β1 may be the main downstream molecule that mediated the pro-fibrotic function of MIF. Interestingly, we also found that high concentrations of TGF-β1 significantly increased MIF expression at the mRNA and protein levels, suggesting that TGF-β1 accumulation in the lesion sites can in turn induce MIF, which may form a positive feedback loop promoting joint capsule inflammation and fibrosis. However, the related mechanisms remain to be further clarified.

Our study has some limitations. We cultured primary joint capsule fibroblasts for *in vitro* experiments. Although the method of fibroblast culture in this research has been approved by many previous reports, fibroblast identification can be improved with more advanced technologies. We mainly focused on the effect of inflammatory factors on the expression of TGF-β1 only in the early stage of contracture development, trying to intervene PTJC at the source. Other people's work has been reported that TGF-β1 levels are chronically elevated in patients and animals with PTJC 10-12, 39, 40. Given that the change and role of MIF in human joint capsule fibrosis remains unclear and our finding that high TGF-β1 concentrations induced MIF expression, human joint capsule tissue or joint capsule fibroblast should be used to detect the effect of MIF on TGF-β1 expression, as well as whether MIF expression increases again in the later stage due to the long-term accumulation of TGF-β1.

In conclusion, based on our previous as well as present findings, our data demonstrate that MIF protein levels increase following PTJC, which promotes TGF-β1 expression from joint capsule fibroblasts through interaction with CD74 receptor and subsequent activation of P38 and ERK pathways. TGF-β1 in turn facilitates fibroblast differentiation and MIF production, thus generating a positive feedback loop regulating joint capsule inflammation and fibrosis during PTJC.

## Supplementary Material

Supplementary figures and tables.
