# Supplementary material for: Macrophage migration inhibitory factor regulates joint capsule fibrosis by promoting TGF-β1 production in fibroblasts
Source: Int J Biol Sci. 2021 Apr 29;17(7):1837–50. doi: 10.7150/ijbs.57025 (PMC8120472; doi:10.7150/ijbs.57025)
Supplement: Supplementary file 1 — Supplementary figures and tables. [file ijbsv17p1837s1.pdf]

Supplementary materials

Group	Control group (n=6/time point)					4-IPP group (n=6/time point)				
Treatment	Rat PTJC model									
	Intra-articular injection of vehicle					Intra-articular injection of 4-IPP				
Time point	0 d	1 d	3 d	7 d	14 d	0 d	1 d	3 d	7 d	14 d

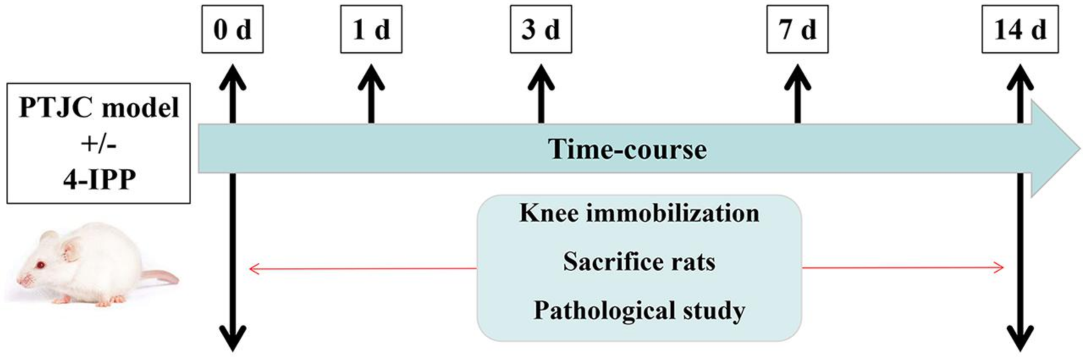

Figure S1. Experimental design and flowchart of the animal experiment.

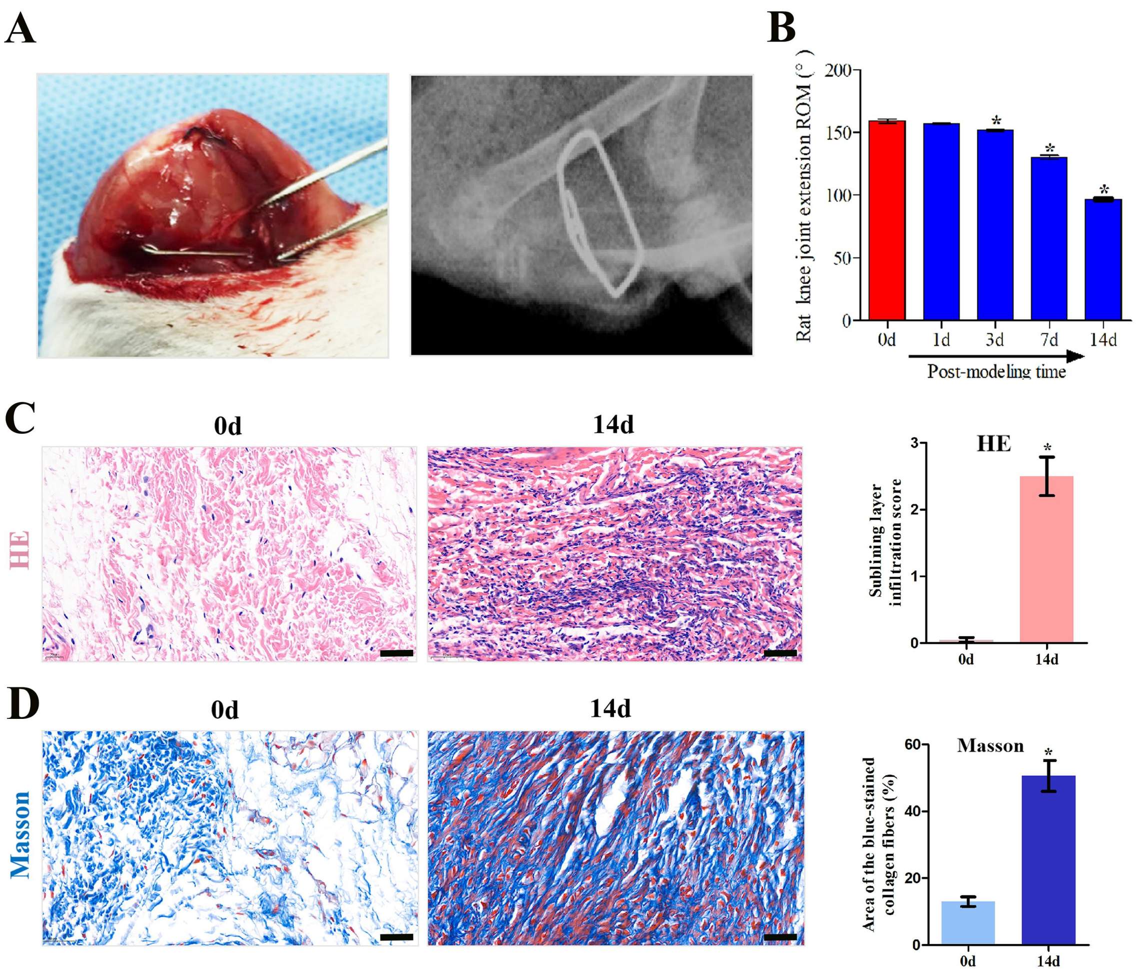

Figure S2. Establishment of the rat knee joint PTJC model.

A, Schematic diagram and X-ray of rat knee joint post-traumatic immobilization. **B,** Measurement of the affected knee passive extension ROM at 0, 1, 3, 7, and 14 days post-induction of PTJC. **C, D,** HE and Masson staining of the injured posterior joint capsule at 0 and 14 days after PTJC. Scale bars, 50 μm. All experiments were conducted independently at least three times. Error bars represent standard deviation. *P < 0.05 compared with 0 d group.

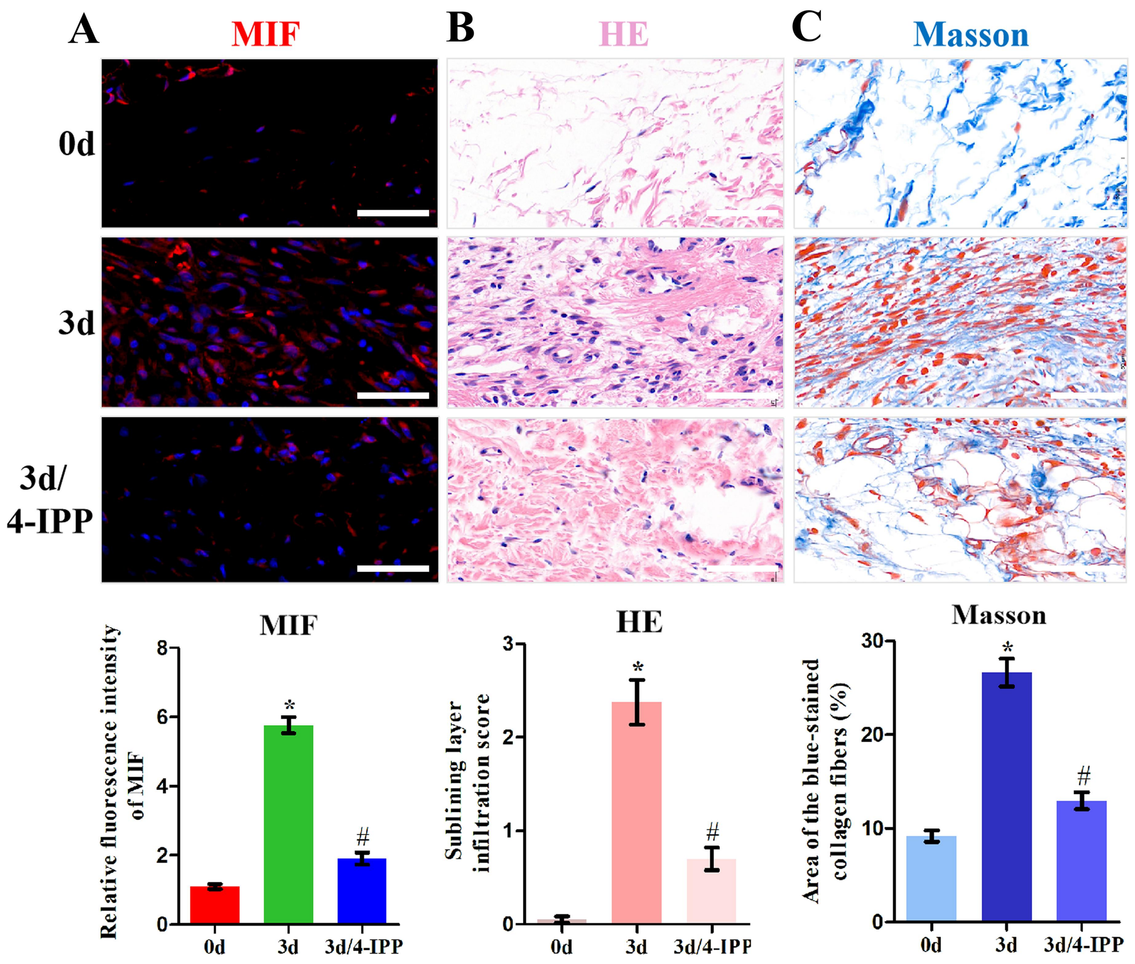

Figure S3. Inhibition of MIF in the lesion area attenuated posterior joint capsule inflammation and fibrosis.

A, Expression of MIF (red) in the posterior joint capsule was assessed via immunostaining at 0 d, 3 d and 3 d after injection of 4-IPP. **B,** HE staining of the posterior joint capsule. **C,** Masson staining of the posterior joint capsule. Scale bars, 50 μm. All experiments were conducted independently at least three times. *P < 0.05 compared with 0 d group. #P < 0.05 compared with 3 d group.

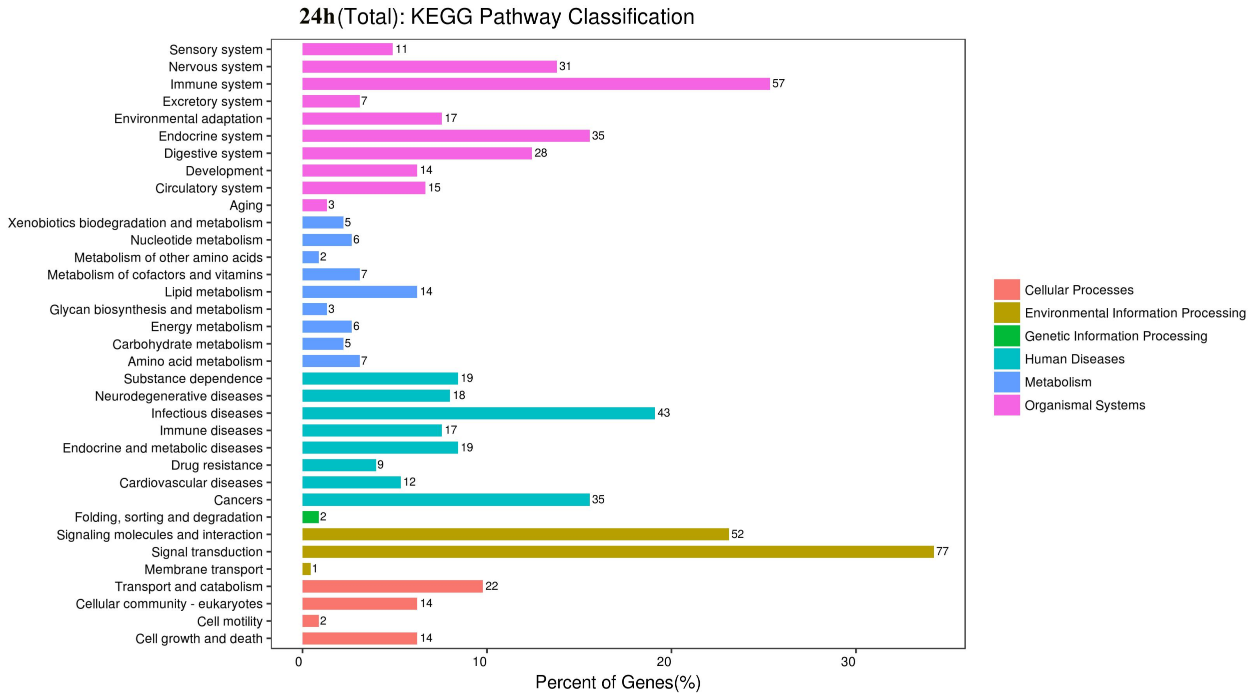

Figure S4. KEGG analysis of all the DEGs after MIF treatment of fibroblasts for 24 h.

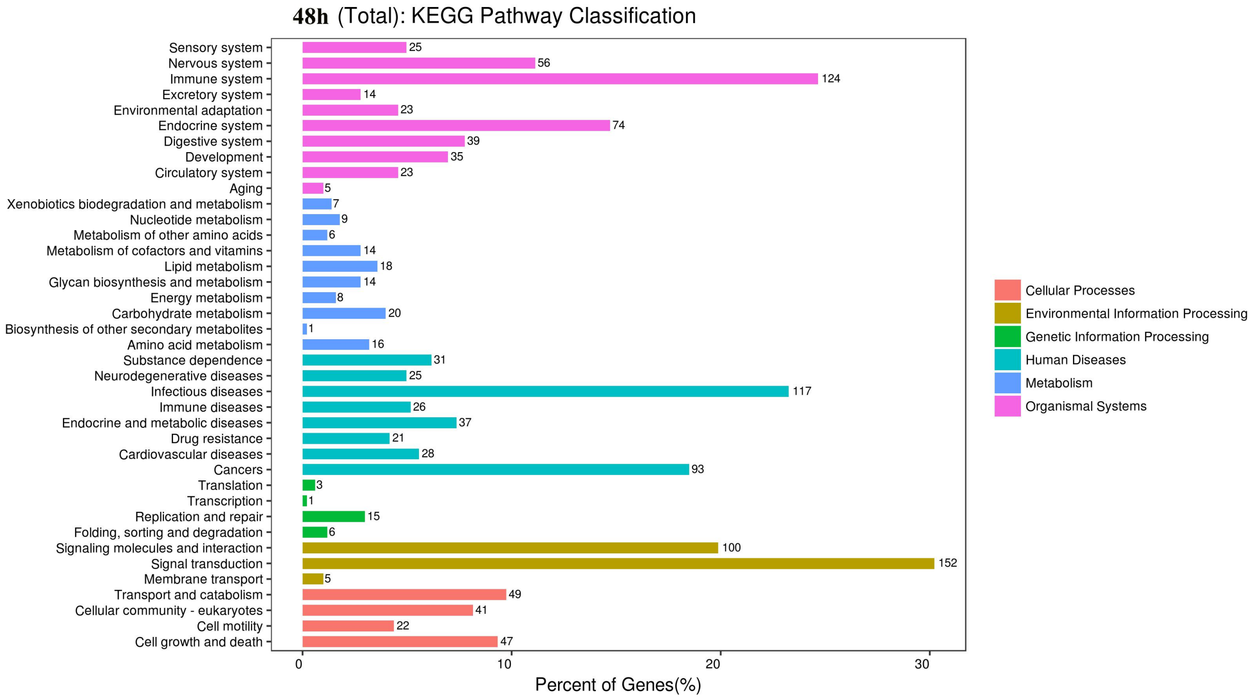

Figure S5. KEGG analysis of all the DEGs after MIF treatment of fibroblasts for 48 h.

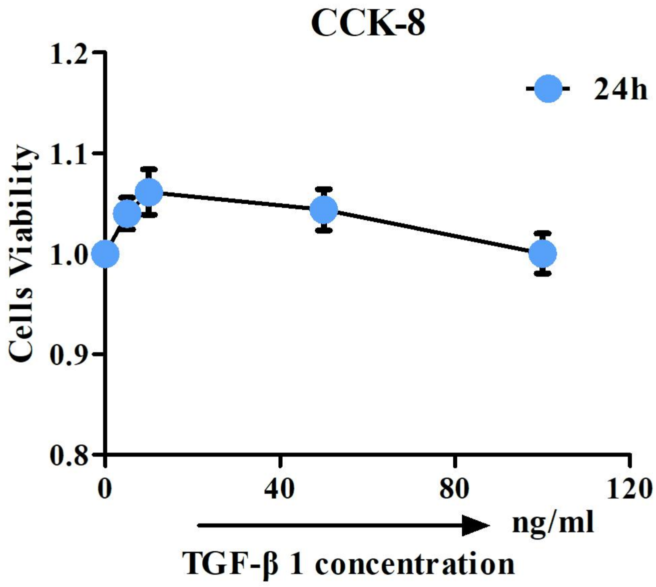

Figure S6. Determination of joint capsule fibroblast viability.

CCK-8 assay was performed following joint capsule fibroblasts treatment with 0–100 ng/mL recombinant TGF-β1 for 24 h. Experiments were conducted independently at least three times. Error bars represent standard deviation.
